# A Novel Cellular Senescence-related lncRNA Signature for Predicting the Prognosis of Breast Cancer Patients

**DOI:** 10.7150/jca.96107

**Published:** 2024-07-02

**Authors:** Fangxu Yin, Wenhao Zhao, Chen Ding, Chong Hou, Song Wang, Chao Sun, Zexia Zhao, Zhanrui Zhang, Fan Ren, Yuying Liu, Xuanguang Li

**Affiliations:** 1 Department of Pediatric Surgery, Tianjin Medical University General Hospital, Tianjin, China.; 2 Department of Lung Cancer Surgery, Tianjin Medical University General Hospital, Tianjin, China.; 3 Department of Emergency medicine, Tianjin Medical University General Hospital, China.; 4 Department of Orthopedic Surgery, Tianjin Medical University General Hospital, Tianjin, China.; 5 Department of Pathology, Nanjing Integrated Traditional Chinese and Western Medicine Hospital Affiliated with Nanjing University of Chinese Medicine, Nanjing, China.

**Keywords:** cellular senescence, lncRNA, breast cancer, immune microenvironment, drug therapy

## Abstract

**Background**: Long non-coding RNA (lncRNA), a crucial regulator in breast cancer (BC) development, is intricately linked with cellular senescence. However, there is a lack of cellular senescence-related lncRNAs (CSRLs) signature to evaluate the prognosis of BC patients.

**Methods**: Correlation analysis was conducted to identify lncRNAs associated with cellular senescence. Subsequently, a CSRL signature was crafted in the training cohort. The model's accuracy was evaluated through survival analysis and receiver operating characteristic curves. Furthermore, prognostic nomograms amalgamating cellular senescence and clinical characteristics were devised. Tumor microenvironment and checkpoint disparities were compared between low-risk and high-risk groups. The correlation between these signatures and treatment response in BC patients was also investigated. Finally, functional experiments were conducted for validation.

**Results**: A signature comprising nine CSRLs was devised, which demonstrated adept prognostic capability in BC patients. Functional enrichment analysis revealed that tumor and immune-related pathways were predominantly enriched. Compared to the low-risk group, the high-risk group could benefit more from immunotherapy and certain chemotherapeutic agents. The expression of the 9 CSRLs was validated through in vitro experiments in different subtypes of BC cell lines and tissues. AC098484.1 was specifically verified for its association with senescence-associated secretory phenotypes.

**Conclusion**: The CSRLs signature emerges as a promising prognostic biomarker for BC, with implications for immunological studies and treatment strategies. AC098484.1 has potential relevance in the treatment of BC cell senescence, and these findings improve the clinical treatment levels for BC patients.

## 1. Introduction

BC is the most common cancer, and the leading cause of tumor-associated morbidity and mortality in women 1. In 2020, BC became the second most commonly diagnosed cancer worldwide, second only to lung cancer. Although treatments such as targeted therapy, radiation and chemotherapy are evolving, more than 7.7 million women have a survival time of only five years after diagnosis 2. Therefore, the identification of predictive markers, prognostic factors and new therapeutic targets for BC is of importance for clinical management.

Cellular senescence refers to a state of permanent cell growth arrest in response to various damaging stimuli 3. It is a multifaceted process. On the one hand, senescent cells suppress tumor development by inhibiting the proliferation of harmful cells while on the other hand, they can secrete senescence-associated secretory phenotypes (SASP), including interleukin-6 (IL-6), interleukin-1α (IL-1α) and interleukin-8 (IL-8) 4-6, which promote tumor growth via immunosuppression and inflammation 7. SASP can also play the opposite role by promoting senescent cells through autocrine or paracrine peri cells to undergo senescence, thereby suppressing tumor cells 8,9. Cellular senescence plays an important role in BC development 10. However, it has not been fully established whether cellular senescence is a potential biomarker for clinical prognosis and treatment outcomes.

LncRNAs are non-coding RNAs over 200 nucleotides in length that do not encode for proteins, but regulate gene expressions at multiple levels. They have been shown to affect cell growth and senescence through multiple pathways. LncRNAs play important regulatory roles in BC biological processes, such as proliferation, invasion, and metastasis 11-13. They have become a major focus of cellular senescence research, a process in which they are actively regulated 14-16. For instance, lncRNA 7SL inhibits p53 translation, promotes cell cycle progression and suppresses cellular senescence as well as autophagy, while its silencing promotes p53 translation and accumulation, contributing to cell cycle arrest and cellular senescence in cervical cancer cells 17. Lnc-IL7R inhibits lipopolysaccharide-induced inflammatory responses and expressions of pro-inflammatory factors. Moreover, lnc-IL7R negatively regulates inflammatory TNF-α and IL-8 cytokines 18. The significance of cellular senescence in the tumor immune microenvironment has not been fully characterized. For example, LncRNA Lethe, which is induced by nuclear factor κB (NF-κB) or glucocorticoid receptor agonists, is involved in inhibition of NF-κB-induced signaling and inflammation 19. The lncRNA HOTAIR regulates NF-κB activation by inhibiting the activities of Iκ-Bα, inducing NF-κB activation and NF-κB target gene expressions in response to DNA damage 20. Therefore, the roles of cellular senescence in the BC immune microenvironment should be further explored.

Elucidation of the role of LncRNA in cellular senescence is important for its potential impact in aging and related diseases as well as its use as a biological marker for prevention, screening, diagnosis and prognosis of aging-related diseases. Therefore, we constructed a model of CSRLs, analyzed and assessed the relevance of prognostic and clinicopathological features in BC patients, and explored the value of BC patients in tumor immune infiltration and immunotherapy. Our findings show the regulatory mechanisms of CSRLs in BC, which may inform clinical decisions.

## 2. Materials and Methods

### 2.1 Patients and Datasets

We downloaded standardized RNA-seq data from The Cancer Genome Atlas Breast Cancer (TCGA-BRCA; https://portal.gdc.cancer.gov/). A total of 1085 BC patients were included in this study. Complete clinical and prognostic information were downloaded from TCGA. Subsequently, we selected a total of 1,022 patients with complete clinical information and an overall survival (OS) of more than 30 days. Using the R package "caret", 1022 BC patients were randomly divided into training (512 patients) and validation (510 patients) cohorts at a 1:1 ratio. Table 1 presents the distribution of clinical characteristics among patients in different groups. A total of 279 cellular senescence-related genes (CSRGs) were acquired from the Cellular Senescence Database (https://genomics.senescence.info/cells/). The site is a genetic database developed to facilitate the study of cellular senescence by integrating a large number of data sets to allow for a systems biology analysis of cellular senescence. We collected cancer and paraneoplastic tissues from six BC patients. This study was approved by the Ethics Committee of Tianjin Medical University, and each patient provided written informed consent.

### 2.2 Identification of CSRLs

Correlation between CSRGs and lncRNAs was analysed. Pearson correlation coefficient > 0.4 and p < 0.001 as thresholds for identification of CSRLs. The “ggalluvial” in R was used to obtain Sankey plots.

### 2.3 Establishment and Validation of the CSRLs Prognostic Signature

For the identified 1391 CSRLs, first, we used univariate Cox regression analysis and LASSO regression to obtain CSRLs that were associated with the prognosis of BC patients, the LASSO regression method used the “glmnet” package for R. Then used multivariate Cox regression analysis to establish risk scores for construction of CSRLs prognostic models. Patient risk scores were calculated using the following formula: Risk score = Σ (each gene's expression × corresponding coefficient). We employed linear regression analysis to assess the survival relationship between the low-risk and high-risk groups. The accuracy of the model was evaluated using the receiver operating characteristic (ROC) curve. We analyzed the differences in OS between the low-risk and high-risk groups using the "survival" package in R.

### 2.4 Construction of Nomogram and Calibration

Risk scores were combined with clinical pathological characteristics, including age, clinical stage, T stage, N stage, and M stage, we used the "rms" package in R to construct a nomogram for predicting 1-, 3-, and 5-year survival outcomes in BC patients. The accuracy of the nomogram was determined using calibration curves.

### 2.5 Functional Enrichment Analyses of CSRLs Signature

Based on the median risk score, BC patients were assigned into low-risk and high-risk groups. The “limma” package was employed to identify the differentially expressed genes (DEGs). Gene Set Enrichment Analysis (GSEA) was employed to identify pathways that were enriched in both groups, utilizing the gene set “c2.cp.kegg.v7.4.symbols.gmt”. And Gene Ontology (GO) and Kyoto Encyclopedia of Genes and Genomes (KEGG) analyses were employed using "clusterProfiler" package.

### 2.6 Estimation of Tumor Microenvironment

Single-sample gene set enrichment analysis (ssGSEA) was used to analyze the abundance of immune cell infiltration in BC. The "estimate" algorithm was used to calculate stromal, immune, and ESTIMATE scores for patients.

### 2.7 Roles of the Predictive Signature in Predicting Clinical Treatment Outcomes

To determine the roles of predictive signals in BC treatment responses, we calculated the half-maximal inhibitory concentrations (IC50) of common targeted and chemotherapeutic agents for BC treatment using "pRRophetic" package. The Wilcoxon signed rank test was used to compare IC50 values between high-risk and low-risk groups.

### 2.8 RNA Extraction, Reverse Transcription, and Quantitative Real-time PCR (qRT-PCR)

MCF-7, T47D, SK-BR-3, MDA-MB-231 human BC cells and MCF-10A human breast epithelial cells were cultured in MEM medium + 10% FBS + 1% pyruvate (purchased from gibco) to above 90% confluence. Extract RNA from the above cell lines and BC tissues according to the instructions provided with the Feijer Biotechnology RNA Extraction Kit. cDNA was reverse transcribed using a high volume cDNA kit (Thermofisher). GAPDH was used as an endogenous control. The primers were purchased from Shanghai Biotechnology Co. The sequences are shown in Supplementary Table 1. We silenced AC098484.1 expression by using siRNA-mediated silencing. siRNA duplexes targeting AC098484.1 were transfected into tumor cells using Lipofectamine 2000 (Invitrogen, USA) in serum-free medium according to the manufacturer's instructions.

### 2.9 Senescent Cell Assay

Using β-galactosidase staining (senescence β-galactosidase staining kit), analysis of senescent cells was conducted. Fresh SA-β-Gal staining solution was prepared according to the manufacturer's instructions (YEASEN), then BC cells, transfected or not with siRNA, were seeded into 6-well plates for staining. Images of stained cells were captured under a microscope, and the percentage of senescent cells was quantified by Image J software, based on the percentage of SA-β-Gal-positive cells in randomly selected areas (n=3).

### 2.10 Statistical Analysis

All statistical analyses were performed using R (version 4.3.1). Statistical significance was set at P < 0.05.

## 3. Results

### 3.1 CSRLs with Prognostic Values in BC

**Figure 1:**
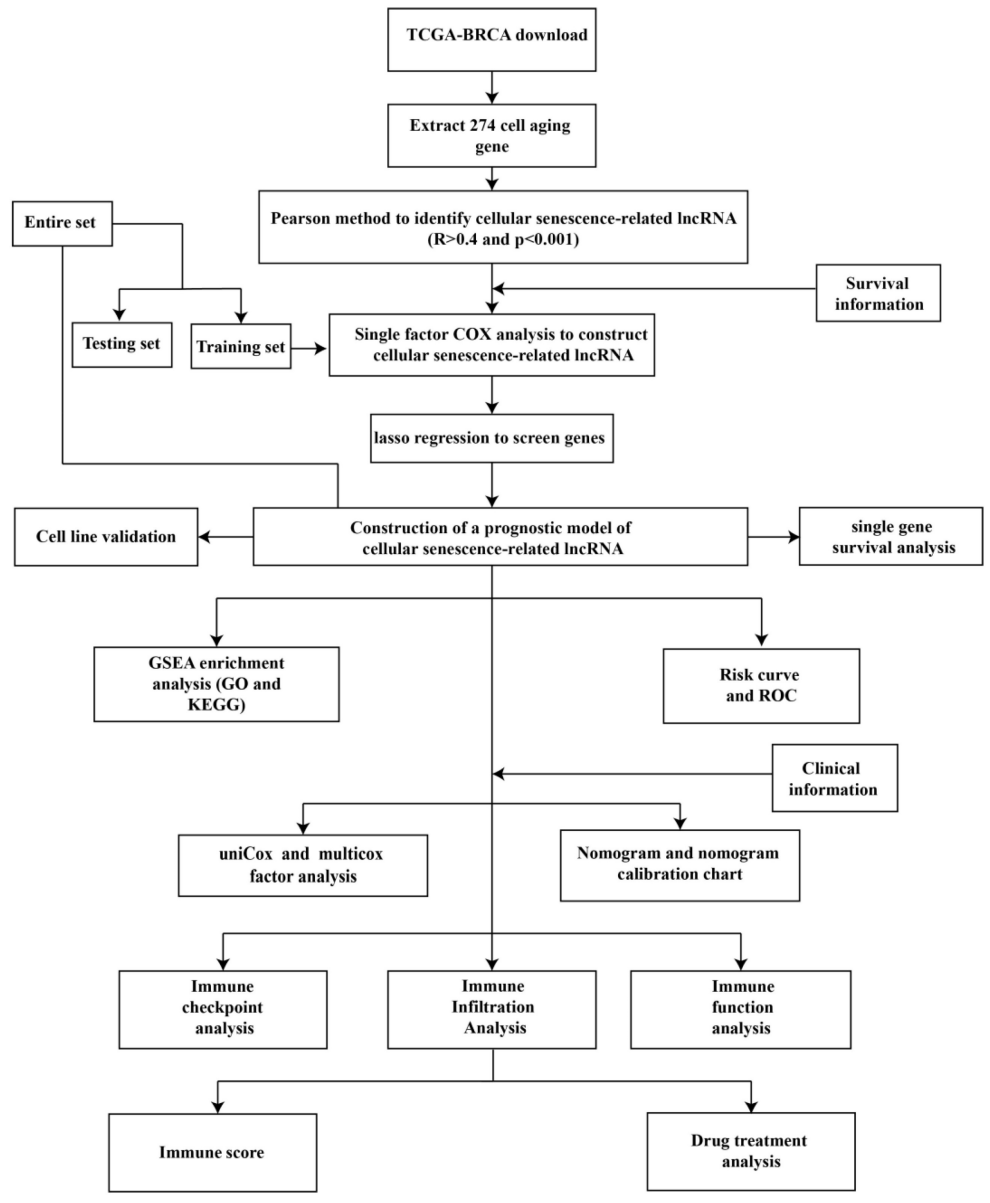
The flow chart of this study.

**Figure 2:**
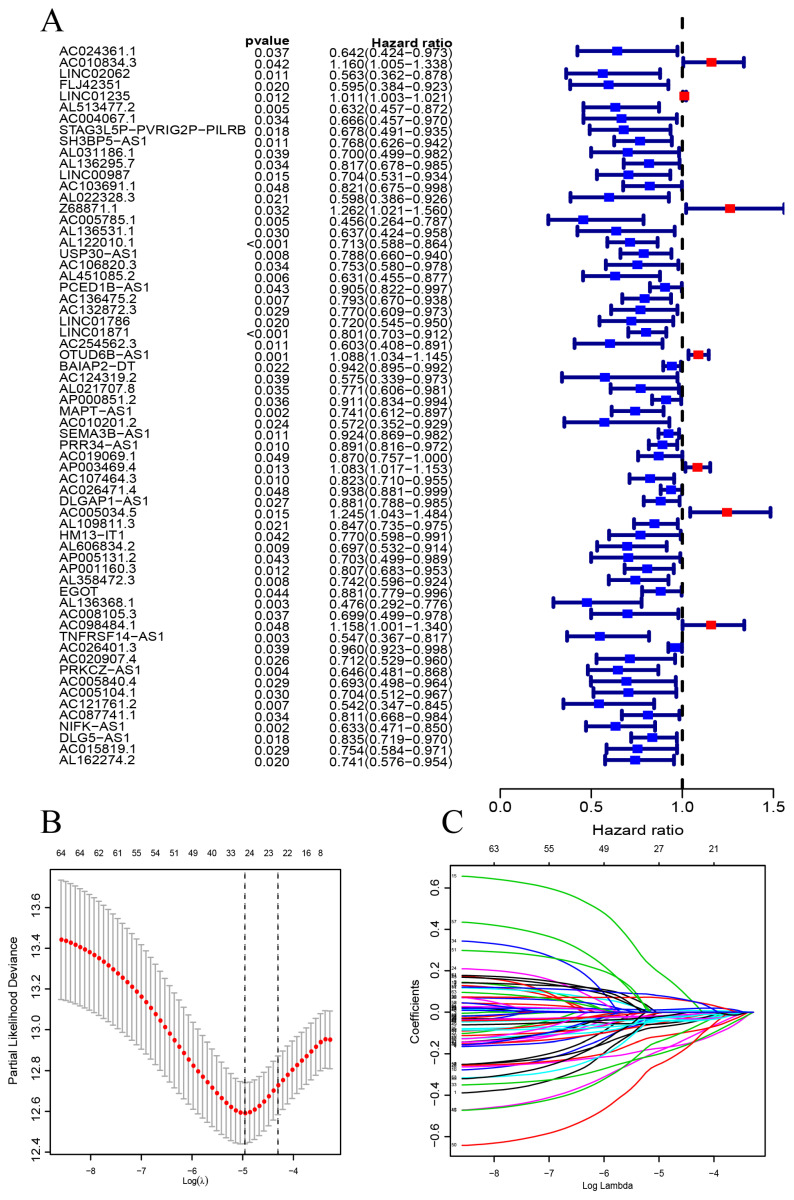
The construction of a prognostic signature in BC patients. (A) Forest plots showing the results of the univariate Cox regression analysis between the 64 CSRLs and OS of BC. (B) 24 CSRLs were selected by the LASSO regression model according to minimum criteria. (C) The coefficient of CSRLs were calculated by LASSO regression.

Figure 1 is a flow chart of our research. First, we screened 279 CSR genes, of which 274 genes had their expression data in TCGA-BRCA (Supplementary Table 2). Then, 1391 CSRLs were identified by Pearson correlation analysis (|R2| > 0.4, p < 0.001). Sixty four lncRNAs were screened by univariate Cox regression, and their expressions correlated with patient outcomes, indicating that they had a prognostic value for BC (Figure 2A). We ultimately identified 7 lncRNAs associated with poor prognosis in BC (HR > 1) and 57 lncRNAs associated with favorable prognosis in BC (HR < 1).

### 3.2 Prognostic CSRLs Signature

**Figure 3:**
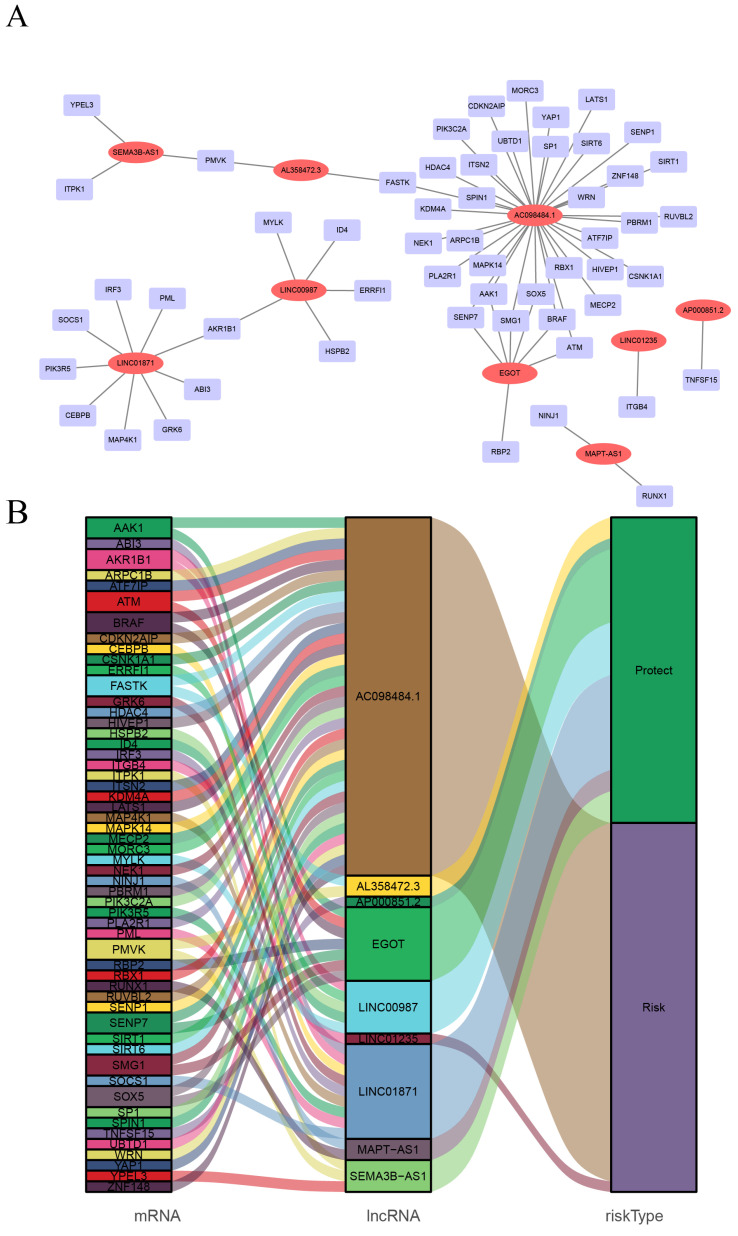
Screening of prognostic CSRLs in BC. (A) A prognostic co-expression network of the 9 CSR lncRNAs-mRNAs. (B) The Sankey diagram of the relationship between lncRNA and mRNA.

**Figure 4:**
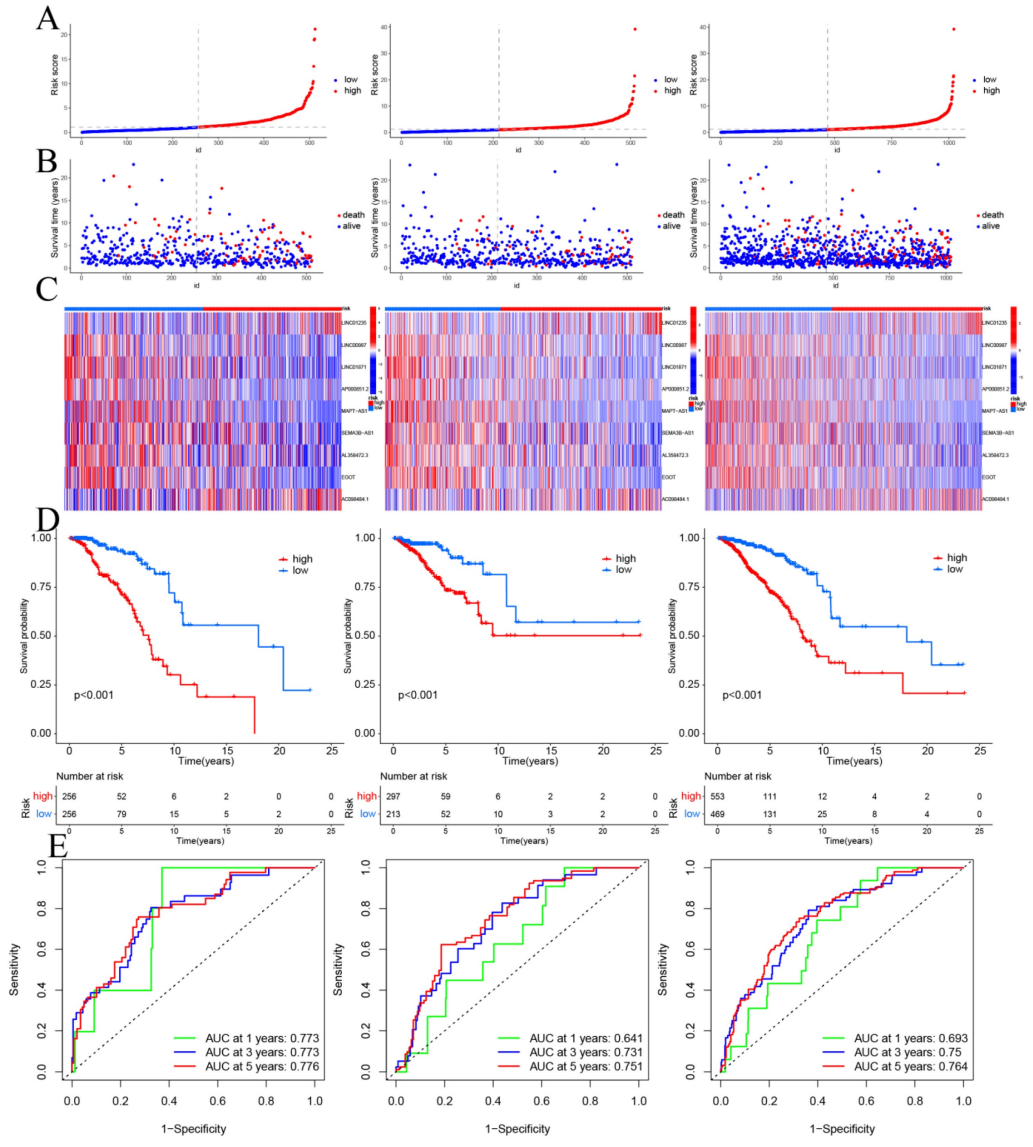
Prognosis values of the 9 CSRLs signatures in the train, test, and entire cohorts. The distribution of (A) risk scores, (B) survival time and survival status, (C) heat maps of 9 lncRNAs expression. (D) Kaplan-Meier survival curves of overall survival of BC patients between low-risk and high-risk groups in the train, test, and entire cohorts, respectively. (E) The AUC of the ROC curve shows the accuracy of the prognostic model in the train, test, and entire cohorts.

CSRLs with P <0.05 in univariate analysis were included in the LASSO regression. LASSO regression identified 24 CSRLs (Figure 2B,C). LASSO results were then included in multivariate Cox models to create risk scores. Nine CSRLs were identified by multivariate Cox proportional risk regression analysis in training cohort. Correlations between expressions of these 9 lncRNAs and CSR genes are shown in Figure 3A. Two lncRNAs (LINC01235 and AC098484.1) were identified as independent adverse prognostic factors, while the remaining lncRNAs (LINC00987, LINC01871, AP000851.2, MAPT-AS1, SEMA3B-AS1, AL358472.3, and EGOT) were independent favorable prognostic factors (Figure 3B). The risk scoring formula is as follows: (0.3913×LINC01235)+(-0.7395×LINC00987)+(-0.6437×LINC01871)+(-0.4137×AP000851.2)+(-0.5358×MAPTAS1)+(-0.3491×SEMA3BAS1)+(-0.4013×AL358472.3)+(-0.3817×EGOT)+(-0.6652×AC098484.1). Risk scores for each patient were calculated based on formula. Patients in the training cohort were divided into low-risk and high-risk groups using the median risk score as the cutoff point. The distributions of risk score, survival status, and nine lncRNAs expression between the low-risk and high-risk groups in training, validation and all cohorts are shown in Figure 4A-C. Kaplan-Meier survival curves revealed significant differences in OS between low-risk and high-risk groups in training, validation and all cohorts (p < 0.001, Figure 4D), indicating that the newly developed signal effectively predicted survival. The ROC curves of the training cohort indicated AUC values of 0.773, 0.773, and 0.776 for 1-, 3-, and 5-year OS rates respectively. In the validation cohort, the AUC values were 0.641, 0.731, and 0.751 for 1-, 3-, and 5-year OS rates, while for the all cohort, the AUC values were 0.693, 0.750, and 0.764 for 1-, 3-, and 5-year OS rates, respectively (Figure 4E). Kaplan-Meier survival analysis showed the OS of 9 CSRLs at high and low expression (Supplementary Figure 1). We performed a survival analysis of the risk model by using different physiological and clinical factors (e.g., age, stage, T, N, M). The Kaplan-Meier survival curves showed that OS was better in almost all low-risk groups compared to the high-risk group (Supplementary Figure 2). Stage Ⅳ (p=0.723), T4 (p=0.624), N3 (p=0.240), M1 (p=0.723) were not statistically significant, which we believe is due to the lower number of patients, but overall the high-risk group had worse OS.

### 3.3 Construction of a Predictive Nomogram

**Figure 5:**
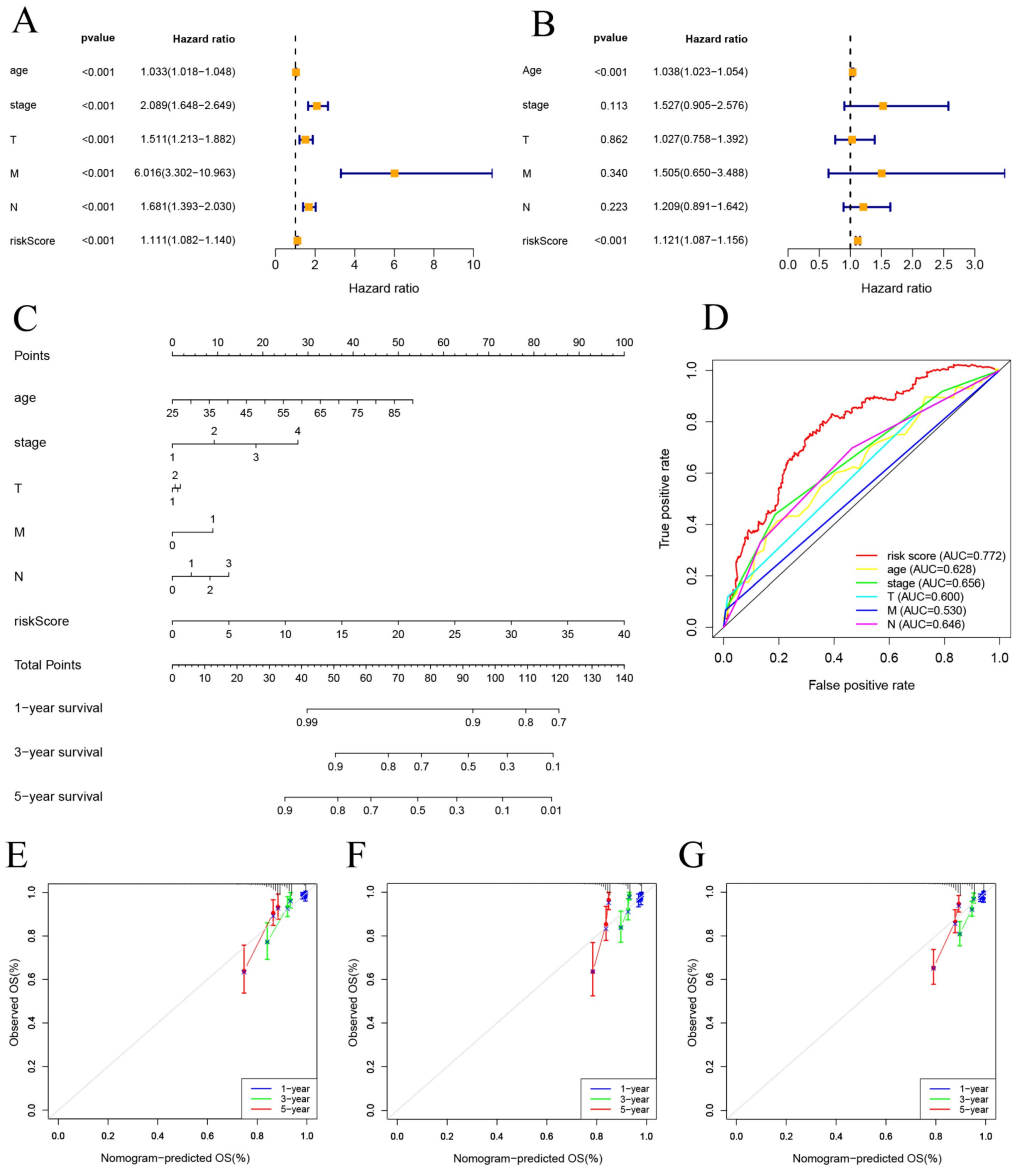
Construction of nomogram and calibration. (A, B) The hazard ratio (HR) and 95% confidence interval of risk score and clinical features were calculated using the univariate and multivariate Cox regression analysis. (C) Clinical prognostic nomogram was developed to predict 1-, 3-, and 5-year survival. (D) Time-dependent ROC curve analyses for predicting OS at 5 years by risk score age, stage, T stage (tumor size), M stage (distant metastasis), N stage. (E-G) Calibration curves showing nomogram predictions for 1-year, 3-year, and 5 year survival in the train, test, and all cohorts.

Univariate and multivariate Cox regression analyses were performed on risk score and clinical features. The results indicate that both the risk score and age are independent factors impacting the prognosis of BC patients (Figure 5A, B). And a nomogram using a combination of CSRLs Signature with other clinicopathological factors was constructed to estimate 1-, 3-, and 5-year survival outcomes in BC patients (Figure 5C). A time-dependent ROC curve for 5-year OS was generated, and an AUC value of 0.772 for risk score was significantly higher than that for age, clinical stage, T stage, M stage, and N stage. These findings imply that the established nomogram had a good ability to predict BC survival outcomes (Figure 5D). Calibration plots of nomogram at 1, 3, and 5 years showed that mortality rates estimated by the nomogram were close to actual mortality rates in training, validation and all cohort (Figure 5E-G).

### 3.4 Pathway Enrichment Analysis

**Figure 6:**
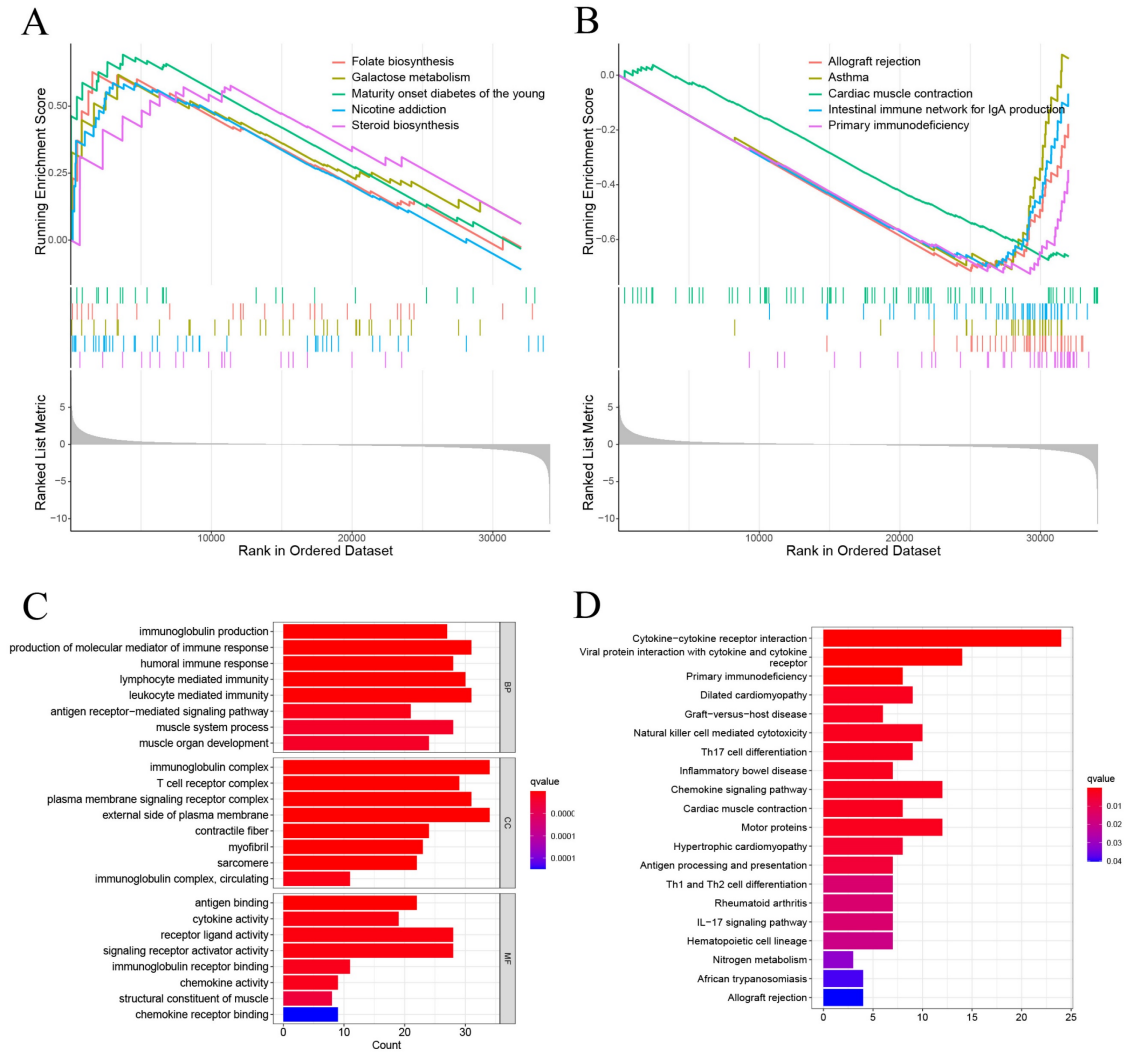
The results of functional analysis based on DEGs between low-risk and high-risk groups. (A, B) The enriched gene terms in GSEA. (C) Column diagrams of Gene Ontology analysis for the DEGs. (D) Column diagrams of KEGG analysis for for the DEGs.

GSEA showed that the DEGs between high-risk and low-risk groups were significantly enriched on Folate biosynthesis, Galactose metabolism, Maturity onset diabetes of the young, Nicotine addicion, and the Steroid biosynthesis in high-risk group (Figure 6A). And Allograft rejection, Asthma, Cardiac muscle contraction, Intestinal immune network for igA production, and Primary immunodeficiency in low risk group (Figure 6B). The GO enrichment analysis revealed that pathways were highly enriched on external side of plasma membrane, immunoglobulin complex, production of molecular mediator of immune response, and leukocyte mediated immunity (Figure 6C). KEGG analysis revealed that DEGs were mostly associated with Cytokine-cytokine receptor interaction, Viral protein interaction with cytokine and cytokine receptor, Primary immunodeficiency, Dilated cardiomyopathy, and Graft-versus-host disease (Figure 6D). These results suggest that the DEGs between high-risk and low risk groups may be associated with the tumor immune microenvironment.

### 3.5 Immune Cell Infiltrations

**Figure 7:**
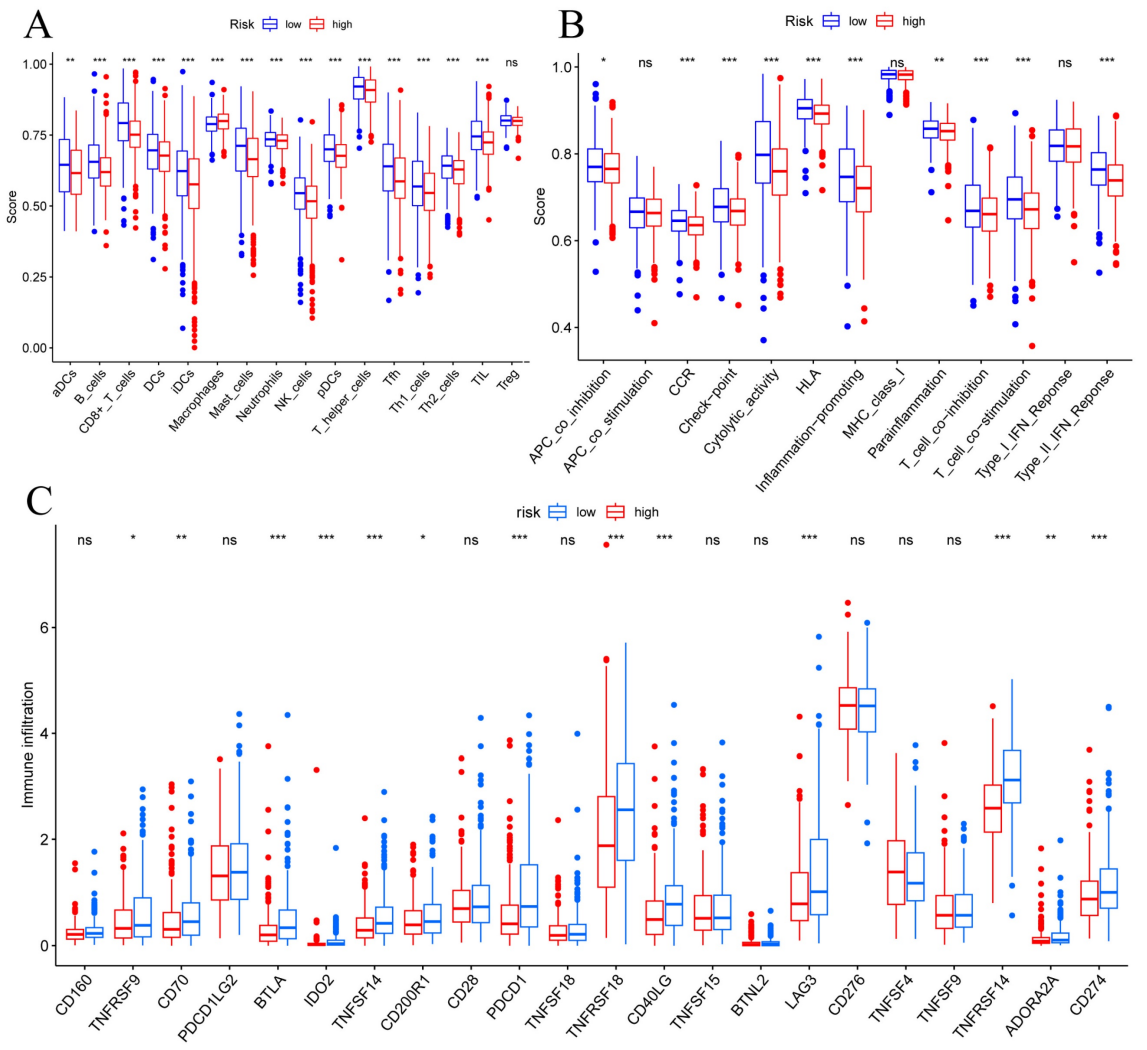
TME, and checkpoint analysis in BC. (A) The box plots of immune cells between the low-risk and high-risk groups. (B) The box plots of immune related pathways between the low-risk and high-risk groups. (C) The box plots of checkpoint related genes between the low-risk and high-risk groups. ^Ns^p ≥ 0.05, *p < 0.05, **p < 0.01, ***p < 0.001.

To determine correlations between risk scores of immune cells and functions, we callculated the score of different immune cell subpopulations. Activated dendritic cells (aDCs), B cells, CD8+ T cells, DCs, immature dendritic cells (iDCs), macrophages, mast cells, neutrophils, natural killer cells, plasmacytoid dendritic cells (pDCs), T helper cells, T follicular helper cells (Tfh), T helper type 1 (Th1) cells, T helper type 2 (Th2) cells and tumor-infiltrating lymphocytes (TIL) were significantly different in the high- and low-risk groups (Figure 7A). Antigen-presenting cell (APC) co-inhibition, chemokine receptor (CCR), checkpoint, cytolytic activity, human leukocyte antigen (HLA), inflammation promotiong, parainflammation, T cell co-inhibition, T cell co-stimulation, and type II IFN responses were significantly different in the high- and low-risk groups (Figure 7B). Therefore, we assessed the expression of checkpoint genes, found that most (e.g., TNFRSF9, CD70, LAG3, CD274, etc.) were highly expressed in the low-risk group (Figure 7C). These findings suggest that immune cell infiltrations of BC was closely correlation of risk score.

### 3.6 Correlations Between the Predictive Signature and BC Therapy

**Figure 8:**
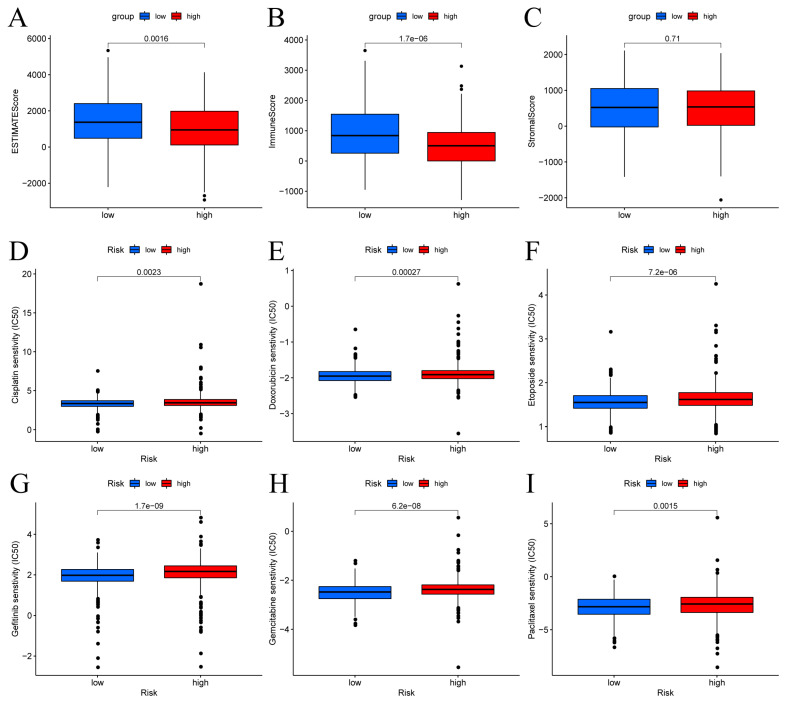
Immune score and drug sensitivities between low-risk and high-risk groups in BC. (A-C) Differences in ESTIMATE scores, immune scores, and stromal scores between the different risk score groups. Boxplots depict differences in estimated IC50 levels of (D) cisplatin, (E) doxorubicin, (F) etoposide, (G) gefitinib, (H) gemcitabine, and (I) paclitaxel between risk score groups.

The patients in the high-risk group exhibited lower estimate scores, immune scores, but there was no difference between the two groups in stromal scores (Figure 8A-C). Using the R package "pRophetic," the IC50 values of chemotherapeutic drugs commonly used in the treatment of BC were compared, and the results showed that patients with low risk scores were more sensitive to cisplatin, doxorubicin, etoposide, gefitinib, gemcitabine and paclitaxel (Figure 8D-I).

### 3.7 Expression of Nine Prognostic CSRLs Signature in BC

**Figure 9:**
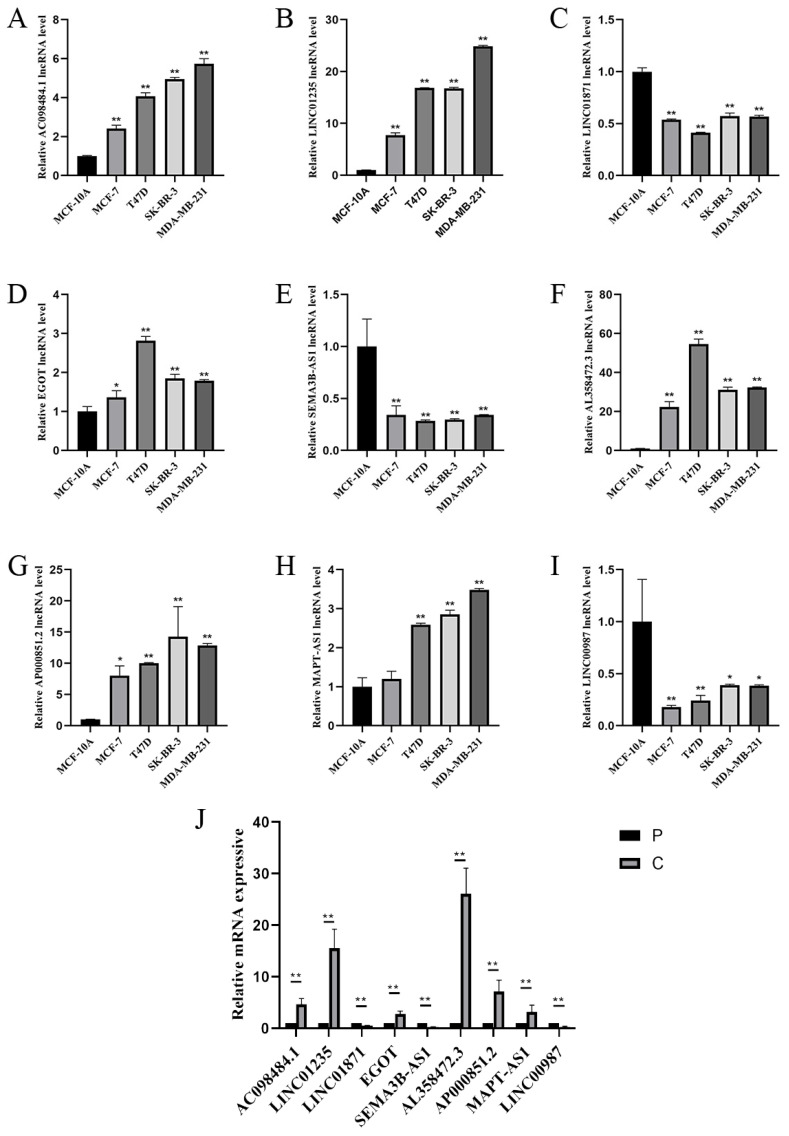
(A-I) RT-PCR validation of 9 CSRLs using MCF-7, T47D, SK-BR-3, MDA-MB-231 human BC cells and MCF-10A human breast epithelial cells. And (J) RT-PCR validation of 9 CSRLs using human BC and paraneoplastic tissues. Expression of nine prognostic factors in BC: AC098484.1, LINC01235, LINC01871, EGOT, SEMA3B-AS1, AL358472.3, AP000851.2, MAPT-AS1, and LINC00987. *p < 0.05; **p < 0.01; ns, non-significant.

To study the expression of LINC01871, LINC01235, LINC00987, EGOT, SEMA3B-AS1, AL358472.3, AC098484.1, AP000851.2 and MAPT-AS1, BC cell lines (MCF-7, T47D, SK-BR-3, MDA-MB-231) and normal breast cell lines (MCF-10A), and in BC tissues and paraneoplastic tissues. The expression of LINC01871, LINC01235, LINC00987, EGOT, SEMA3B-AS1, AL358472.3, AC098484.1, AP000851.2 and MAPT-AS1 is shown in Figure 9 A-I. Among them, patients with high expression of AC098484.1, LINC01235, EGOT, AL358472.3, and AP000851.2 had a poor prognosis. Patients with low expression of LINC01871, SEMA3B-AS1, and LINC00987 had a poor prognosis. This differs from the results of the online analysis. In particular, the validation of MAPT-AS1 expression in the MCF-7 cell line showed no statistically significant difference, while the validation of AL358472.3, AP000851.2, and EGOT expression contradicted the results of the online survival analysis. The expression trends of the above 9 CSRLs in BC tissues are consistent with those in the cell lines (Figure 9J).

### 3.8 Knockdown of AC098484.1 Promotes Cellular Senescence in BC

**Figure 10:**
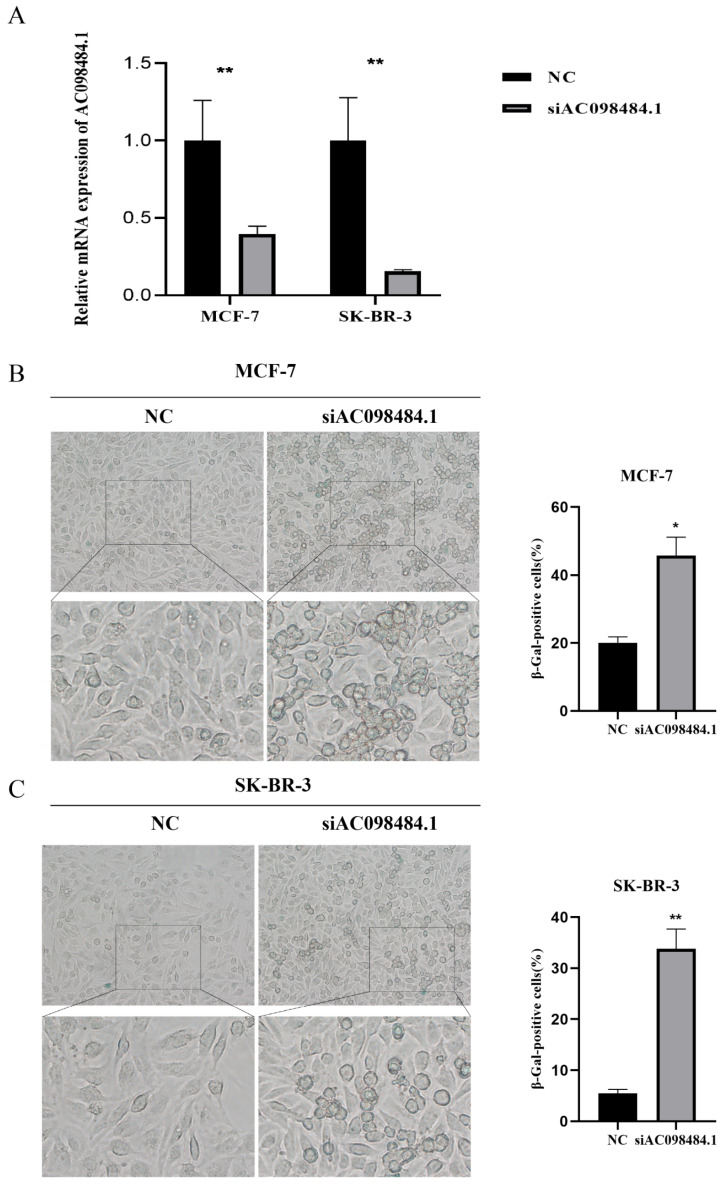
Knockdown of AC098484.1 promotes senescence in BC cells. (A) Reduced expression of AC098484.1 in MCF-7 and SK-BR-3 cells. (B) AC098484.1 was knocked down in MCF-7 cells, and its effect on cellular senescence was analyzed using SA-β-gal staining. (C) AC098484.1 was knocked down in MCF-7 cells, and its effect on cellular senescence was analyzed using SA-β-gal staining. *p < 0.05; **p < 0.01; ns, non-significant.

SA-β-gal levels were measured using a Senescence β-Galactosidase Staining Kit. Compared to the control group, both the percentage and intensity of SA-β-gal-positive cells significantly increased in the AC098484.1 knockdown group (Figure 10 A-C).

## 4. Discussion

Globally, BC is the leading cause of morbidity and mortality among women 21, which necessitates the identification of effective prognostic biomarkers. Some mRNAs and lncRNAs play an important role as prognostic molecular markers for malignancy 22,23. By combining various biomarkers for survival prediction of disease, we can individualize patient treatment and improve survival outcomes.

Cellular senescence is an anti-proliferative program that leads to permanent cell growth arrest 24, and with increasing research on cancer suppression mechanisms, stopping tumor development by inducing tumor cell senescence is gaining attention. Lleonart *et al.* 25 reported that cellular senescence can achieve anti-tumor effects. In BC cells, MALAT1 down regulation blocked G1 phase and inhibited cell growth as well as proliferation 26.

Studies have reported on BC-related lncRNA signaling, including autophagy 27 and iron death 28, but there are no signaling studies on CSRL. Therefore, we constructed a model using 9 CSRLs (LINC01235, AC098484.1, LINC00987, LINC01871, AP000851.2, MAPT-AS1, SEMA3B-AS1, AL358472.3, and EGOT). This signature could clearly distinguish between the groups, suggesting the models' accuracy.

LINC01235, AC098484.1, LINC00987, LINC01871, AP000851.2, MAPT-AS1, SEMA3B-AS1 and EGOT have been studied in BC and other cancers. LINC01235 is an independent prognostic factor in BC patients 29. Hypoxia-related LINC01235 affects the prognostic outcomes for BC patients and may be a potential target for cancer therapy 30. In patients with clear cell renal cell carcinoma, AC098484.1 was associated with autophagy and was established to be a potential prognostic marker 31. Previous findings on LINC00987, MAPT-AS1 and LINC01871 suggest that they can advance immunotherapy in BC patients 32,33, consistent with our findings. AP000851.2 and SEMA3B-AS1 are involved in stemness regulation of BC stem cells and are novel prognostic factors for BC patients 34. EGOT is involved in regulating the expressions of eosinophil protein transcripts, which affects the prognostic outcomes for BC patients and is a potential biomarker 35. There are no corresponding studies on AL358472.3, and further studies are needed to verify whether it is a promising predictive marker. The area under the ROC curve of the model corresponded to survival rates of 0.773, 0.773, and 0.776 at 1, 3, and 5 years, respectively, a result that indicates the good predictive performance of the model and helps to promote individualized treatment of BC patients. The 9 CSRLs were found to be markedly related to clinical factors in both univariate analyse, LASSO regression and multifactorial analyse, followed by the prognostic model related to clinical factors, and the calibration curve showed good discrimination and accuracy of the model, and we speculate that it may be a potential predictive tool for BC patients.

The most important contribution of this study is elucidation of the relationship between the risk score and tumor immune microenvironment. Subsequent ssGSEA analysis revealed that infiltration abundance of macrophages was higher in the high-risk group. Macrophages are important innate immune cells that maintain tissue homeostasis, defend against pathogens and are associated with BC infiltrations and adverse outcomes 36. The low-risk group was correlated with the abundance of lymphocyte infiltration, suggesting that low-risk patients can better fight against tumors through immune defenses than high-risk patients and that T-cell-based therapies are a top priority in BC clinical practice and immunotherapy 37. These findings link CSRLs signature to immune infiltrations in BC, and CSRLs may be targets for combination therapy with immune checkpoint inhibitors. CSRL can regulate various cellular senescence pathways, including telomere shortening, DNA damage response, and cell cycle regulation 38. The mechanisms of action of some chemotherapy and targeted therapy drugs induce apoptosis or proliferation inhibition of tumor cells by affecting these cellular senescence pathways. Therefore, the expression levels of CSRLs may affect the sensitivity of tumor cells to these drugs. Xu *et al.* conducted whole transcriptome sequencing of BC specimens, screening out the lncRNA EGOT, and experimental validation showed that appropriately regulating EGOT may be a novel synergistic strategy to enhance paclitaxel sensitivity in cancer treatment 39. For other lncRNAs within CSRL, no information related to drug sensitivity was retrieved. Our findings also indicate that low-risk patients exhibit higher sensitivity to the targeted therapeutic drug (gefitinib), in addition to chemotherapeutic agents (cisplatin, paclitaxel, etoposide, docetaxel and gemcitabine). Therefore, low-risk patients can benefit from a combination of immunotherapy, targeted therapy and chemotherapy, which can lead to more favorable and personalized treatments for BC patients.

The close correlation between lncRNA expression and prognosis holds significant implications for understanding the molecular mechanisms of aging. Firstly, lncRNAs have emerged as critical regulatory factors in gene expression and cellular processes, playing pivotal roles in various biological functions, including pathways associated with aging such as cellular senescence, inflammation, and genomic stability. By elucidating the specific lncRNAs associated with prognosis, our findings reveal the intricate regulatory networks involved in the pathophysiology of aging. Furthermore, our discoveries may pave the way for identifying novel biomarkers to assess individual risk profiles and tailor personalized interventions to promote healthy aging and longevity. Integrating lncRNA expression profiles into predictive models for assessing age-related health outcomes enables clinicians and researchers to better stratify individuals based on their susceptibility to age-related diseases and implement targeted interventions to optimize healthy lifespan and quality of life. Compared to previous BC prediction models related to cellular senescence, we performed effective experimental validation of the constructed model 40,41. Zhang *et al.* developed a prognostic model based on six cellular senescence genes, and the AUC values of this model were 0.69, 0.74, and 0.74 for 1-, 3-, and 5-year OS, respectively 42. Our model has higher predictive accuracy for 1-, 3-, and 5-year survival predictions. We validated the expression of nine CSRLs in ER-positive, HER2-enriched, and triple-negative BC cell lines (MCF-7, T47D, SK-BR-3, MDA-MB-231) through in vitro experiments. Compared to normal BC epithelial cells MCF-10A, we found that AC098484.1, LINC01235, EGOT, AL358472.3, and AP000851.2 were highly expressed in different BC cell line subtypes. Furthermore, comparison with online analysis data revealed that high expression of these genes correlated with poor prognosis in patients, while low expression of LINC01871, SEMA3B-AS1, and LINC00987 was associated with poor prognosis. This was consistent with the malignancy levels of the different cell line subtypes. However, the expression validation of MAPT-AS1 in the MCF-7 cell line was not statistically significant, and the validation of AL358472.3, AP000851.2, and EGOT expressions contradicted the results of the online survival analysis. This highlights the importance of relying on real experimental data rather than solely on online data for analysis.

Next, we analyzed the expression of nine CSRLs in BC and paraneoplastic tissues and found trends similar to those in cell lines. Ultimately, we concluded that high expression of AC098484.1, LINC01235, EGOT, AL358472.3, AP000851.2, and MAPT-AS1 was associated with poor prognosis, while low expression of LINC01871, SEMA3B-AS1, and LINC00987 was associated with poor prognosis. To understand the relationship with cellular senescence, we selected one significantly trending lncRNA, AC098484.1, for SASP-related experimental validation. Through siRNA-mediated knockdown of AC098484.1, we observed a significant increase in both the percentage and intensity of SA-β-gal positive cells in the knockdown group compared to the control group. This indicated increased senescence in BC cells MCF-7 and SK-BR-3, demonstrating that inhibiting AC098484.1 plays a crucial role in the senescence of BC cells. These findings are expected to improve clinical treatment levels and enhance the prognosis for BC patients.

However, this study has several limitations. First, the data used in this study were from a single data source, which may lead to some bias in the results, and we searched through the GEO database and unfortunately did not find a dataset that matched the CSRLs we studied, probably due to a shortage of external data caused by the relatively few directions of this study in BC, but in the future data from different cohorts and datasets will be used for this model. Secondly, due to insufficient funding, we could not realize the staining images of the 9 CSRLs in actual BC tissues, but we did a preliminary validation of our results by RT-PCR experiments. Further molecular and in vivo validation will be performed to elucidate the role of 9 CSRLs in BC cellular senescence. In the future, we will delve deeper into exploring the potential regulatory roles of CSRLs in TME-related signaling pathways, aiming to further enhance our understanding of their mechanisms in tumor development and treatment.

In conclusion, we developed a prognostic model associated with cellular senescence in BC consisting of 9 CSRLs (LINC01235, AC098484.1, LINC00987, LINC01871, AP000851.2, MAPT-AS1, SEMA3B-AS1, AL358472.3, and EGOT). The model newly identified immune-related lncRNAs in BC patients. AC098484.1 has potential relevance in the treatment of BC cell senescence, and these findings improve the clinical treatment levels for BC patients.

## Supplementary Material

Supplementary figures and tables.

## Figures and Tables

**Tabel 1:** Clinical pathological parameters of patients with breast cancer

Characteristics	Overall	Training cohort	Validation cohort	P
	(n = 1022)	(n = 512)	(n = 510)	
	No. of patients (%)	No. of patients (%)	No. of patients (%)	
**Age**				0.648
≤65	736(72.0)	372(72.7)	364(71.4)	
>65	286(28.0)	140(27.3)	146(28.6)	
**Pathologic Stage**				0.086
Ⅰ	187(18.3)	93(18.2)	94(18.4)	
Ⅱ	583(57.0)	297(58.0)	286(56.1)	
Ⅲ	232(22.7)	107(20.9)	125(24.5)	
Ⅳ	20(2.0)	15(2.9)	5(1.0)	
**T Stage**				0.906
T1	278(27.2)	143(27.9)	135(26.5)	
T2	581(56.8)	288(56.3)	293(57.5)	
T3	128(12.6)	65(12.7)	63(12.4)	
T4	35(3.4)	16(3.1)	19(3.7)	
**N Stage**				0.581
N0	480(47.0)	245(47.7)	235(46.1)	
N1	345(33.8)	178(34.8)	167(32.7)	
N2	109(10.6)	49(9.6)	60(11.8)	
N3	71(6.9)	31(6.1)	40(7.8)	
Nx	17(1.7)	9(1.8)	8(1.6)	
**M Stage**				0.070
M0	847(82.8)	423(82.6)	424(83.1)	
M1	20(2.0)	15(2.9)	5(1.0)	
Mx	155(15.2)	74(14.5)	81(15.9)	
