# Supplementary material for: A Novel Cellular Senescence-related lncRNA Signature for Predicting the Prognosis of Breast Cancer Patients
Source: J Cancer. 2024 Jul 2;15(14):4700–16. doi: 10.7150/jca.96107 (PMC11242350; doi:10.7150/jca.96107)
Supplement: Supplementary file 1 — Supplementary figures and tables. [file jcav15p4700s1.pdf]

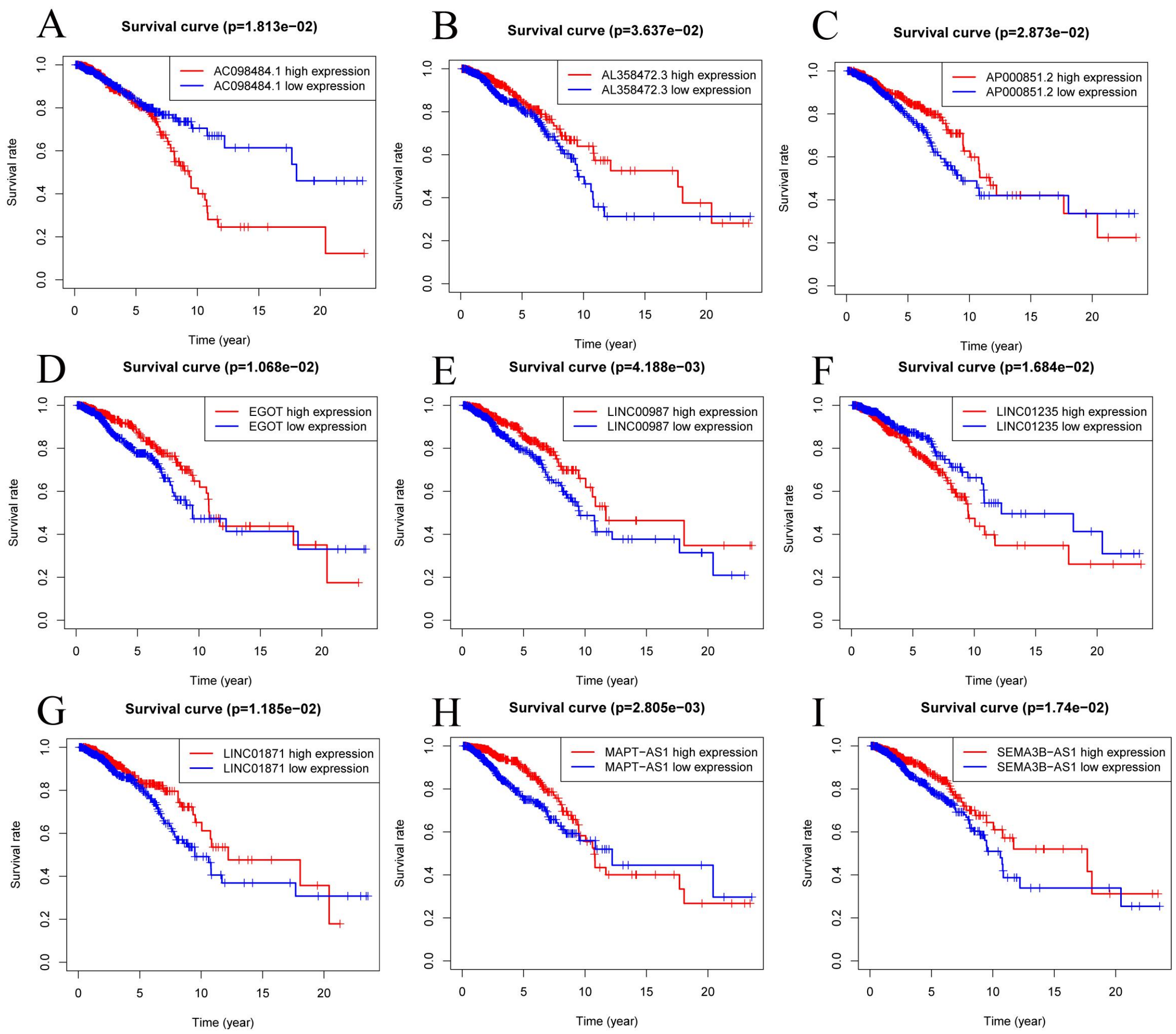

Supplementary Figure 1 Survival curves of CSRLs in BC patients in the low-risk and high-risk groups: (A) AC098484.1, (B) AL358472.3, (C) AP000851.2, (D) EGOT, (E) LINC00987, (F) LINC01235, (G) LINC01871, (H) MAPT-AS1, and (I) SEMA3B-AS1 (P < 0.001).

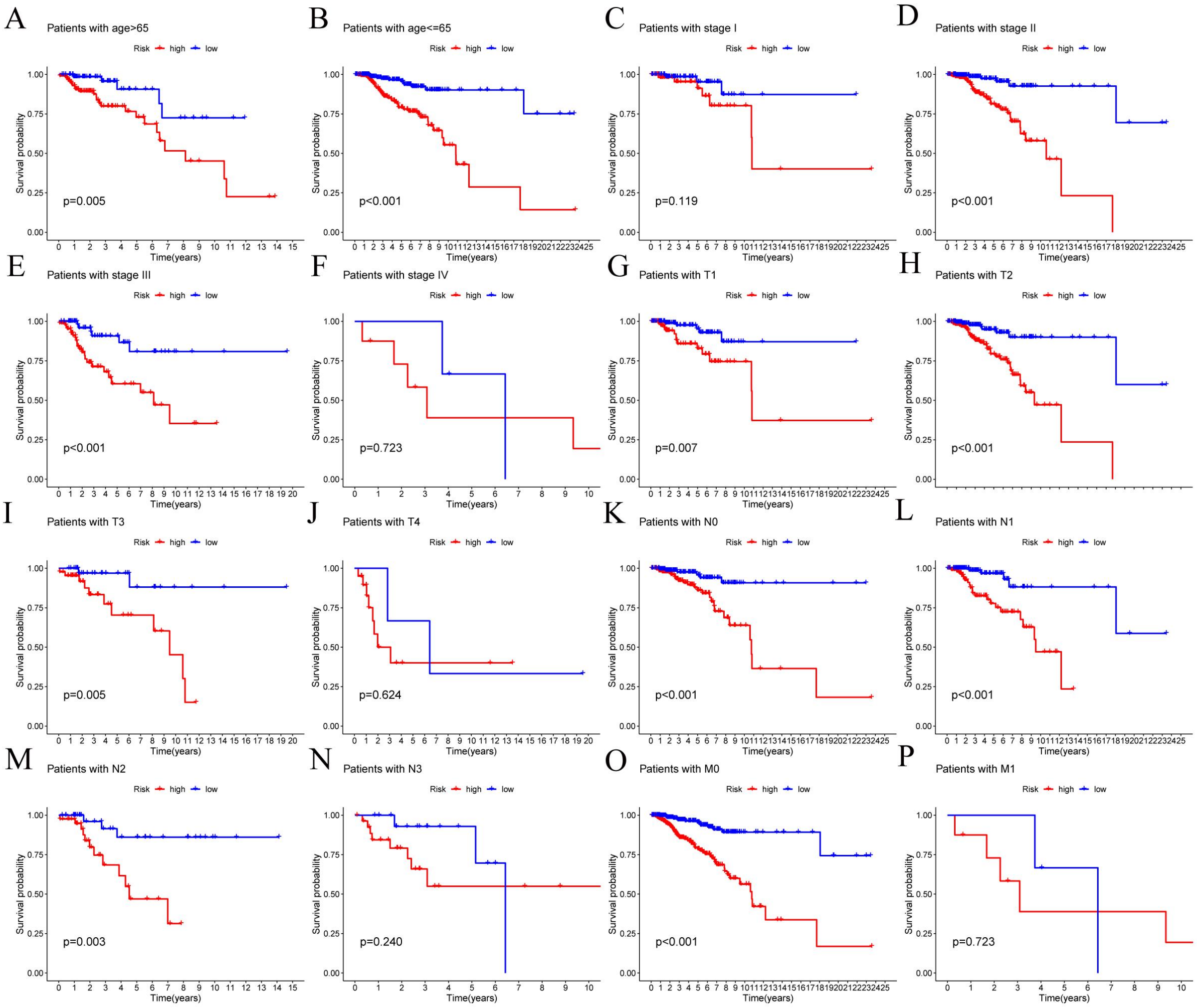

Supplementary Figure 2 Kaplan–Meier survival curves of low-risk and high-risk groups sorted by different clinicopathological variables: (A, B) Age, (C-F) TNM Stage, (G-J) T stage, (K-N) N stage and (O, P) M stage.

Supplementary Table 1 The primer sequences

LINC01871	
Forward Primer	CTGCTGATGTGTGGTGCTTA
Reverse Primer	TCGGCCTTTGGTAGTGTGACT
LINC01235	
Forward Primer	AAGCCGAAGGGTCTCTCACG
Reverse Primer	CCACGTCAAGGCTCCTCAATC
LINC00987	
Forward Primer	GACGACGCACAATGCAAAGAC
Reverse Primer	ACTGGGGAAACCTCCTCCAAAT
EGOT	
Forward Primer	TGGCACGATGGTAAGAGTCCA
Reverse Primer	CAGGTTGGGCTGGATTTCAGG

SEMA3B-AS1	
Forward Primer	AACCTCTCCCTCCACAGGTTG
Reverse Primer	GGAGCTCCGTCCTGTCTGTAA
AL358472.3	
Forward Primer	TGCCTGACTAGACCCTGTTCC
Reverse Primer	CAGAAAAGGGTAGCTGGGTGA
AC098484.1	
Forward Primer	AGTATCATCGGAGTGGCTGAG
Reverse Primer	CCAGGTTTGGGGAGAGTCAAC
AP000851.2	
Forward Primer	TGGTCAAAACTGGGCAGAGTG
Reverse Primer	ACTGTGGCGAGGTGATATGGA
MAPT-AS1	
Forward Primer	GTGTATGCCCTCACAGGTCAC
Reverse Primer	GGGTTACTCTCCCGCACACTA

Supplementary Table 2

A list of cellular senescence-related genes.

ACLY	DLX2	KDM4A	PAK4	SMARCB1
AAK1	DHX9	KDM5B	PBRM1	SMURF2
ABI3	DPY30	KIAA1524	PCGF2	SNAI1
ADCK5	DUSP3	KL	PDCD10	SOCS1
AKR1B1	DUSP16	KSR2	PDIK1L	SOD1
AGT	E2F1	LATS1	PDZD2	SORBS2
AKT1	EHF	LEO1	PDPK1	SOX2
ALOX15B	ENDOG	LGALS3	PEBP1	SPIN1
AR	EPHA3	LIMA1	PEX19	SOX5
ARPC1B	ERRFI1	LIMK1	PIAS4	SP1
ASF1A	ETS1	MAGEA2	PIK3R5	SPOP
ASPH	ETS2	MAGOH	PIK3C2A	SRC
ATF7IP	EWSR1	MAD2L1	PIM1	SREBF1
ATM	FASTK	MAGOHB	PLA2R1	SRSF1
AURKA	EZH2	MAP2K1	PKM	STAT5B

AXL	FBXO31	MAP2K3	PML	STK32C
BAG3	FOXM1	MAP2K2	PNPT1	STK40
BHLHE40	FOS	MAP2K6	PMVK	SUPT5H
BCL6	FOXO3	MAP3K6	POT1	SYK
BLK	FXR1	MAP2K7	POU5F1	TACC3
BLVRA	G6PD	MAP4K1	PPM1B	TERC
BMI1	GAPDH	MAP3K7	PPM1D	TBX2
BRAF	GKN1	MAPK12	PRMT6	TERF2
BRD7	GATA4	MAPKAPK5	PRKCH	TERT
BRCA1	GNG11	5-Mar	PRKCD	TFAP4
BTG3	GLB1	MAPK14	PROX1	TFDP1
C11orf31	GRK6	MAST1	PRPF19	TGFB1I1
CAV1	HDAC4	MATK	PSMB5	TLR3
CBX7	HDAC1	MCL1	PTRF	TMSB4X
CBX8	HEPACAM	MDH1	PTTG1	TNFSF13
CCND1	HJURP	MCRS1	PSMD14	TNFSF15
CDK1	HIVEP1	MECP2	RAD21	TOP1
CDK18	HK3	MOB3A	RAF1	TP63
CDK2AP1	HMGB1	MMP9	RB1	TPR
CDK6	HRAS	MORC3	RBP2	TP53
CDK4	HSPA5	MORF4	RBX1	TRIM28
CDKN1A	HSPB2	MXD4	RNASEL	TRPM8
CDKN1C	ID1	MVK	RPS6KA6	TXN
CDKN1B	ID4	MYC	RSL1D1	TXNIP
CDKN2A	IGFBP1	MYLK	RUNX1	UBTD1
CDKN2AIP	IFNG	NADK	RUVBL2	TYK2
CDKN2B	IGFBP3	NANOG	SENP1	VENTX
CENPA	IGFBP6	NDRG1	SENP2	USP1
CEBPB	IGFBP5	NEK1	SENP7	VEGFA

CHEK1	IL1A	NEK4	SERPINE1	WNT16
CKB	IL8	NEK6	SFN	WNT2
CPEB1	ING1	NFE2L2	SIK1	WRN
CSNK1A1	ING2	NINJ1	SGK1	WT1
CTNNAL1	IRF3	NOTCH3	SIN3B	XAF1
CSNK2A1	IRF5	NOX4	SIRT1	WWP1
CXCL1	IRF7	NR2E1	SIRT6	YAP1
DDB2	ITPK1	NTN4	SIX1	YPEL3
CYR61	ITGB4	NUAK1	SLC13A3	ZFP36
DEK	ITPKB	OTX2	SLC16A7	ZMAT3
DGCR8	ITSN2	P3H1	SMARCA4	ZNF148
DHCR24	KCNJ12	PATZ1	SMG1	
